# Low Skeletal Muscle Mass: A Strong Predictive Factor for Surgical Complications After Free Forearm Flap Reconstruction in Oral Cancer Patients

**DOI:** 10.1002/hed.28014

**Published:** 2024-11-27

**Authors:** E. Ansari, N. Carrillo Minulina, M. A. van Beers, R. J. J. van Es, F. J. Dieleman, A. J. W. P. Rosenberg, L. M. Janssen, W. W. Braunius, E. M. Van Cann, R. de Bree

**Affiliations:** ^1^ Department of Head and Neck Surgical Oncology University Medical Center Utrecht Utrecht The Netherlands; ^2^ Department of Oral and Maxillofacial Surgery University Medical Center Utrecht Utrecht The Netherlands; ^3^ Department of Otolaryngology – Head and Neck Surgery University Medical Center Utrecht Utrecht The Netherlands

**Keywords:** body composition, free ulnar forearm flap, oral cancer, oral reconstruction, radial forearm free flap, sarcopenia, skeletal muscle mass

## Abstract

**Background:**

Low skeletal muscle mass (SMM) is a predictive factor for complications in patients undergoing major head and neck cancer surgery. This study aims to identify the predictive value of low SMM for postoperative complications in patients who underwent free forearm flap (FAFF) reconstructions after oral cancer resections.

**Methods:**

A retrospective study was performed with all patients who underwent FFAF between 2003 and 2020 for an oral cavity reconstruction after cancer ablation. Free flap related, any postoperative complications and hospital stay were investigated.

**Results:**

Low SMM was associated with an increased risk of free flap associated complications (OR 2.14; 95% CI 1.02–4.39, *p* = 0.029). Low SMM was associated with severe complications (Clavien–Dindo ≥ III) (OR 1.46; 95% CI 1.20–2.09, *p* = 0.02).

**Conclusions:**

Low SMM is a strong predictive factor for free flap related surgical complications in patients undergoing FAFF reconstruction after resection of oral cancer.

**Abbreviations**
C3: third cervical vertebra
CSMA: cross‐sectional muscle area
FFAF: free forearm flaps
FRFF: free radial forearm free flap
FUFF: free ulnar forearm free flap
HNC: head and neck cancer
HU: hounsfield unit
L3: third lumbar vertebra
Lumbar SMI: lumbar skeletal muscle index
SMM: skeletal muscle mass

## 1 Introduction

Free forearm flaps (FFAF), that is, the free radial forearm flap (FRFF) and free ulnar forearm flap (FUFF), are frequently used free vascularised flaps for reconstruction of large head and neck soft tissue defects following ablative cancer surgery. A number of preoperative risk factors are known to be associated with postoperative complications after free tissue transfer, which include age, gender, tobacco use, diabetes, hypertension, body mass index (BMI) and prior radiotherapy [1].

Sarcopenia, a condition characterized by loss of skeletal muscle mass (SMM) and low muscle strength, has been found to influence both treatment outcomes and survival in head and neck cancer (HNC) patients [2]. Low SMM alone has been associated with a higher incidence of postoperative complications, chemotherapy related toxicity, longer hospital stays and diminished disease‐free and overall survival in HNC patients [3, 4].

Patients with HNC are more likely to develop sarcopenia because of swallowing disorders caused by the localization of the primary tumor, decreased nutritional intake and cancer‐induced catabolism [5]. Some studies found that sarcopenia is a significant independent risk factor for free flap complications, surgical site infection and other postoperative complications in patients with HNC [3, 7, 8, 9].

The aim of this article is to analyze the association of low SMM with free flap related and other postoperative complications in a subgroup of HNC patients who underwent an FFAF reconstruction following resection of an oral cancer. Furthermore, the relationship between low SMM and the duration of hospital stay is investigated.

## 2 Material and Methods

### 2.1 Ethical Approval

The design of this study was approved by the Medical Ethical Research Committee of the University Medical Center Utrecht (approval ID 17‐365/C). All procedures in this study were in accordance with the ethical standards of the institutional and/or national research committee and with the 1964 Helsinki declaration and its later amendments or comparable ethical standards.

### 2.2 Patients and Study Design

A retrospective study was performed of consecutive patients who underwent reconstruction of oral cavity defects with FFAF after resection of a malignancy between 2003 and 2020 at the Departments of Oral and Maxillofacial Surgery, Otorhinolaryngology and Head and Neck Surgery and Head and Neck Surgical Oncology of the University Medical Center Utrecht, Utrecht, the Netherlands. All surgical procedures were performed by experienced microvascular head and neck surgeons. The choice of an ulnar or radial forearm flap was based on the surgeon's preference and experience with raising the specific flap. Patients were included if they had a recent (less than 1 month before surgery) CT or MRI scan of the head and neck. Clinical and demographic data were collected from the medical records. Data collected included age at surgery, gender, BMI, alcohol consumption (defined as drinking more than 2 units alcohol per day), smoking history (categorized as current smoker or having stopped more than 12 months), diagnosis, TNM stage (pathological), localization of the defect, comorbidity as expressed by the Adult Comorbidity Evaluation‐27 (ACE‐27) score, duration of hospital stay and occurrence of postoperative complications Patients with prior chemoradiation therapy to the head and neck were not included. Neither were patients with recurrences or preoperative nasogastric tube insertion included.

All postoperative complications were scored according to the Clavien–Dindo classification of surgical complications [10]. Patients with multiple complications were scored according to their highest grade of complication. Complications with a Clavien–Dindo grade III–V were graded as severe complications.

Postoperative complications specifically related to the free flap were also analyzed and scored. These were categorized as congestion or thrombosis, partial skin paddle necrosis or dehiscence, donor site morbidity and flap failure.

### 2.3 Body Composition Measurement

**FIGURE 1:**
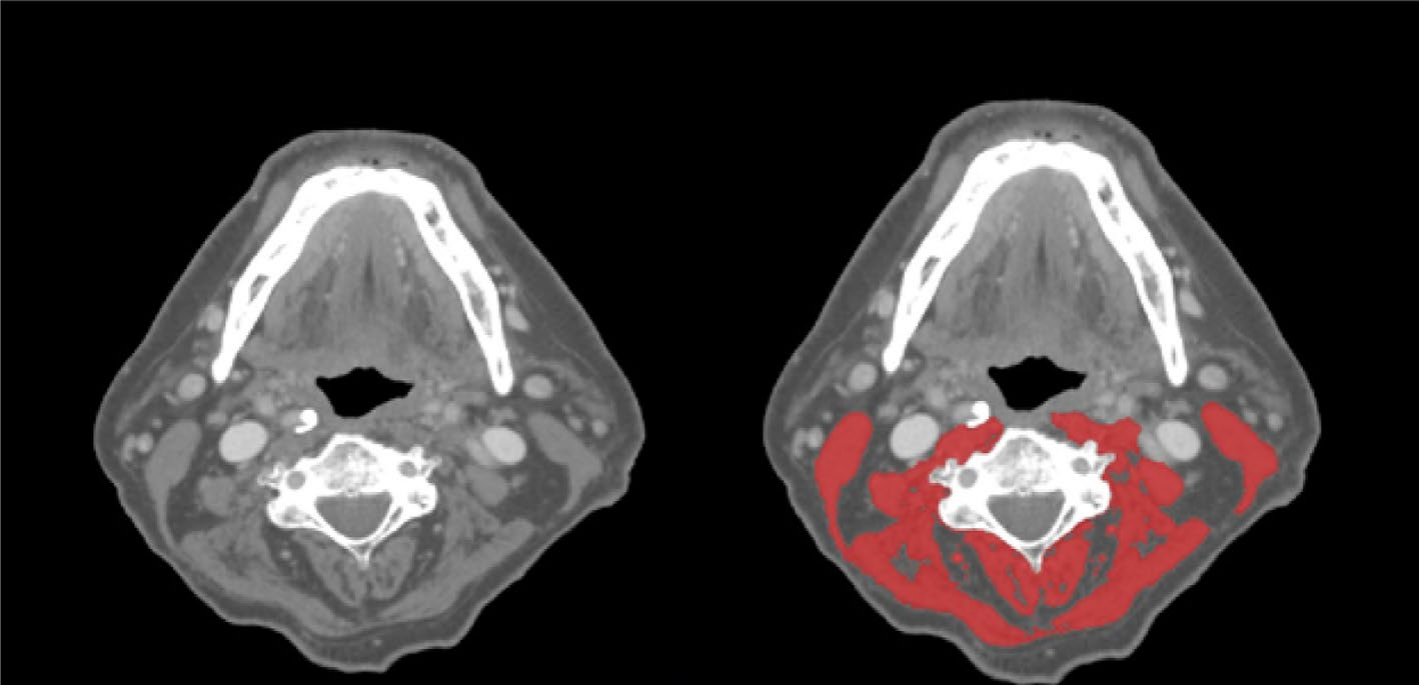
Example of segmentation of skeletal muscle area at the level of the third cervical vertebra. [Color figure can be viewed at wileyonlinelibrary.com]

SMM was measured as muscle cross‐sectional area (CSA) on pre‐treatment CT or MRI imaging of the head and neck area at the level of the third cervical vertebrae (C3). The first axial slide of the imaging when scrolling from cranially to caudally, which showed both transverse processes and the entire vertebral arc, was selected for segmentation of muscle tissue. For CT imaging, muscle area was defined as the pixel area between the radiodensity range of −29 and +150 Hounsfield Units (HU), which is specific for muscle tissue. For MRI, muscle area was manually segmented, and fatty tissue was manually excluded. The CSA was calculated as the sum of the delineated areas of the paravertebral muscles and both sternocleidomastoid muscles. Segmentation of muscle tissue was manually performed using the commercially available software package SliceOmatic (Tomovision, Canada) by a single researcher (NCM) who was blinded for patient outcomes. An example of segmentation at the level of C3 is shown in Figure 1.

CSA at the level of C3 was converted to CSA at the level of L3 using a previously published formula 1 (23). The lumbar skeletal muscle index (LSMI) was calculated by correcting SMM at the level of L3 for squared height as shown in formula 2. Low SMM was defined as a LSMI below 43.2 cm^2^/m^2^, a cutoff value which was determined in a separate cohort of head and neck cancer patients [11].

*Formula 1*:

$$\begin{array}{c} \mathrm{CSA}\;\mathrm{at}\;\text{L3}\;\left({\mathrm{cm}}^{2}\right)=27.304+1.363^{*}\;\mathrm{CSA}\;\mathrm{at}\;\text{C3}\;\left({\mathrm{cm}}^{2}\right)\\ –0.671^{*}\mathrm{Age}\;\left(\text{years}\right)+0.640^{*}\;\text{Weight}\;\left(\mathrm{kg}\right)\\ +26.442^{*}\mathrm{Sex}\;\left(\mathrm{Sex}=1\;\text{for female and}\;2\;\text{for male}\right) \end{array}$$

*Formula 2*:

$$\text{Lumbar}\;\mathrm{SMI}\;\left({\mathrm{cm}}^{2}/m^{2}\right)=\mathrm{CSA}\;\mathrm{at}\;L3/\text{length}\;\left(m^{2}\right)$$

### 2.4 Statistical Analysis

The data analyses were performed using IBM SPSS Statistics version 25.0 (IBM Corp., Armonk, NY, USA). The baseline characteristics were presented as frequencies and percentages. Correlation analysis was performed by use of Pearson's correlation analysis for variables with a normal distribution and Spearman's correlation analysis was used for non‐normally distributed variables. Logistic regression was used for univariate and multivariate analysis of surgical complications. Covariates used in the multivariate analysis were selected based on clinical significance or selected based on statistical significance (*p* < 0.20) in univariate analysis. A test of normality (One sample Kolgomov–Smirnov) was performed for the duration of hospital stay. If hospital stay was normally distributed, an independent sample *t*‐test or a linear regression model was performed if the assumptions of linear regression were met. Statistical significance in this analysis was evaluated at the *p* < 0.05 level using two‐sided tests.

## 3 Results

### 3.1 Patient Characteristics

Descriptive data are presented in Table 1. In total, 174 patients were included. Low SMM was identified in 115 (66.1%) patients. One hundred and thirty‐eight (79.3%) patients underwent a FRFF and 36 (20.7%) patients underwent a FUFF.

**TABLE 1:** Characteristics of patients with low and normal SMM (*N* = 174).

Variables	Low SMM	Normal SMM	*p* ^a^
	*N* = 115	*N* = 59	
	(66.1%)	(33.9%)	
Gender (*n*, %)			
Female	40 (34.8)	23 (39.0)	0.59
Male	75 (65.2)	63 (61.0)	
Mean age (years, SD)	62.6 (10.6)	62.2 (10.6)	0.91
BMI (mean, SD)	24.0 (3.1)	25.5 (3.9)	0.13
Smoker (*n*, %)			0.37
No	37 (32.2)	23 (39.0)	
Current/former	78 (67.8)	36 (61.0)	
Alcohol (*n*, %)			0.05
No	31 (27.0)	15 (25.4)	
> 2 units/day	84 (73.0)	44 (74.6)	
ACE‐27 score (*n*, %)			0.82
Non	31 (27.0)	19 (32.2)	
Mild	49 (42.6)	21 (35.6)	
Moderate	23 (20.0)	13 (22.0)	
Severe	12 (10.4)	6 (10.2)	
Histology (*n*, %)			0.05
Squamous cell carcinoma	115 (100)	57 (96.6)	
Carcinoma ex pleomorphic adenoma	0 (0%)	1 (1.7)	
Rhabdomyosarcoma	0 (0%)	1 (1.7)	
pTNM‐stage (*n*, %)			0.14
I	4 (3.5)	1 (1.7)	
II	38 (33)	14 (23.7)	
III	12 (10.4)	8 (13.6)	
IVa	49 (42.6)	25 (42.4)	
IVb	12 (10.4)	11 (18.6)	
Localization (*n*, %)			0.05
Lip	1 (0.9)	0 (0)	
Floor of mouth	24 (20.9)	11 (18.6)	
Tongue	53 (46.1)	15 (25.4)	
Alveolar ridge	2 (1.7)	1 (1.7)	
Buccal mucosa	14 (12.2)	6 (10.2)	
Retromolar trigone	14 (12.2)	18 (30.5)	
Involving 2 or more sites of oral cavity	7 (6.2)	8 (13.6)	
Free flap type (*n*, %)			0.633
Ulnar forearm flap	25 (21.7)	11 (18.6)	
Radial forearm flap	90 (78.3)	48 (81.4)	
Duration of hospital stay (days, SD)	14.23 (9.16)	14.40 (7.28)	0.92

Abbreviations: ACE‐27, Adult Comorbidity Evaluation‐27; BMI, body mass index.

^a^ Categorical data were analyzed by Fisher exact test. Comparisons of continuous quantitative variables were performed by Mann–Whitney *U*‐test or Student *t*‐test, depending on whether the data were normally distributed.

### 3.2 All Complications

Table 2 shows the non‐flap complications. Clavien–Dindo scores of all postoperative complications are described in Table 3. In total, 117 (67.2%) patients had any postoperative complication, of whom 77 (65.8%) had low SMM.

**TABLE 2:** Type of non‐flap complications in patients with low and normal SMM.

	Normal SMM (%)	Low SMM (%)
Type of non‐flap complication	*N* = 51	*N* = 89
None	18 (3.5)	28 (31.5)
Wound infection	5 (9.8)	10 (11.2)
Postoperative bleeding	4 (7.8)	2 (2.2)
Fistula	5 (9.8)	4 (4.5)
Seroma	1 (2.0)	2 (2.2)
Hematoma	1 (2.0)	3 (3.4)
Chyle leakage	0 (0)	2 (2.2)
Pneumonia	1 (2.0)	8 (9.0)
Gastrointestinal infection	2 (3.9)	0 (0)
Urinary tract infection	0 (0)	1 (1.1)
Fever E.C.I. treated with antibiotics	1 (2.0)	2 (2.2)
Cardiovascular	9 (17.6)	17 (19.1)
Pulmonary embolism	1 (2.0)	2 (2.2)
Stroke	1 (2.0)	1 (1.1)
Delirium	2 (3.9)	7 (7.9)

**TABLE 3:** Clavien–Dindo classification of complications in patients with low and normal SMM.

Clavien–Dindo (CD) Classification (*n*, %)			
	Low SMM	Normal SMM	*p* ^a^
CD I	16 (9.2)	11 (6.3)	
CD II	34 (19.5)	15 (8.6)	
CD III	19 (10.9)	10 (5.7)	
CD IV	4 (2.3)	4 (2.3)	
CD V	4 (2.3)	0 (0)	
Total	77 (44.3)	40 (34.2)	0.91

^a^ Pearson's *χ* ^2^ test, two‐sided.

Forty‐one patients (23.6%) had severe complications (Clavien–Dindo III–V), of whom 27 (65.9%) had low SMM. Four patients (2.3%), all with low SMM, died in hospital within 1 month due to a complication.

The results of the univariate analyses on potential risk factors for any postoperative complications are shown in Table 4. Age, alcohol use, smoking, BMI, ACE‐27 score and SMM were included in the multivariate logistic analysis.

**TABLE 4:** Univariate logistic regression analysis of potential predictors for any postoperative complications.

Complications			
	Univariate analysis		
Variables	OR	95% CI	*p*
Gender			
Male	Ref.		
Female	1.04	0.54–2.01	0.90
Age	1.03	0.99–1.06	0.08
Tobacco	1.37	0.69–2.70	0.17
Alcohol	1.33	0.64–2.78	0.45
BMI	0.95	0.86–1.04	0.24
Low SMM	1.17	0.89–1.64	0.13
ACE‐27			
Non	Ref.		
Mild	0.47	0.13–1.62	0.31
Moderate	1.27	0.18–2.11	0.14
Severe	1.34	0.15–2.11	0.22
Free flap			
Radial forearm flap	Ref.		
Ulnar forearm flap	0.97	0.44–2.11	0.94
Localization (*n*, %)			
Floor of mouth	Ref.		
Tongue	0.97	0.11–1.62	0.24
Lip	0.53	0.21–1.23	0.46
Alveolar ridge	1.04	0.65–2.16	0.31
Buccal mucosa	0.69	0.47–0.89	0.29
Retromolar trigone	0.85	0.19–1.72	0.57
Involving 2 or more sites of oral	1.11	0.2–2.37	0.61
TNM‐stage			
I	Ref.		
II	0.53	0.07–3.98	0.54
III	0.48	0.16–1.42	0.39
IVa	0.35	0.10–1.27	0.21
IVb	1.18	0.40–3.47	0.31

The results of the multivariate analysis are shown in Table 5. Low SMM was not associated with any postoperative complications (OR 1.18; 95% CI 0.58–2.57, *p* = 0.64). Furthermore low SMM was significantly associated with severe postoperative complications (Clavien–Dindo III–V) (OR 1.46; 95% CI 1.20–2.09, *p* = 0.02).

**TABLE 5:** Multivariate logistic regression analysis of potential predictors for any postoperative complications.

Complications			
	Multivariate analysis		
Variables	OR	95% CI	*p*
Age	1.03	1.00–1.06	0.12
Tobacco	1.48	0.68–3.20	0.32
Alcohol	1.16	0.53–2.57	0.72
BMI	0.93	0.84–1.03	0.17
Low SMM	1.18	0.58–2.41	0.64
ACE‐27			
Non	Ref.		
Mild	0.57	0.16–2.10	0.40
Moderate	0.74	0.21–2.59	0.64
Severe	0.54	0.13–2.17	0.38
TNM‐stage			
I	Ref.		
II	0.30	0.03–2.61	0.27
III	0.32	0.09–1.08	0.07
IVa	0.24	0.06–1.21	0.05
IVb	0.99	0.30–3.29	0.99

### 3.3 FFAF Related Complications

FFAF‐related complications are described in Table 6. Complications related to the FFAF occurred in 47 (27.0%) patients, of which 25 (53.2%) occurred in patients with low SMM. Five (3%) patients needed flap revision due to venous thrombosis or arterial occlusion. In 3 (1.7%) patients, the complete flap was lost. In the logistic regression, low SMM was associated with flap related complications (OR 2.14; 95% CI 1.02–4.39, *p* = 0.029).

**TABLE 6:** Free forearm flap related complications in patients with low and normal SMM.

Type of free flap related complication^b^			
	Low SMM	Normal SMM	*p* ^a^
Congestion or thrombosis	2 (4.3)	2 (4.3)	
Flap dehiscence	21 (44.7)	15 (31.9)	
Donor site morbidity	1 (2.1)	3 (6.4)	
Failure	1 (2.1)	2 (4.3)	
Total	25 (53.2)	22 (46.8)	**0.03**

^a^ Pearson's *χ* ^2^ test, two‐sided.

^b^ Only 1 type of free flap complication occurred in patients of this cohort.

### 3.4 Duration of Hospital Stay

Median length of hospital stay was 14.3 days (95% CI 12.99–15.58, SD 8.54). Mean number of days in hospital was similar for patients with or without low SMM (14.40 days, SD 7.23 and 14.23 days, SD 9.05, respectively).

## 4 Discussion

This study assessed risk factors for the occurrence of postoperative complications in patients who underwent reconstruction of head and neck defects with FFAF after resection of a malignant oral cavity tumor. We compared several potential perioperative predictive factors, of which low SMM was significantly likely to cause more postoperative FFAF related complications such as flap dehiscence, flap necrosis, thrombosis and flap failure. Postoperative complications of any type were not significantly higher in patients with low SMM. However low SMM was predictive for the occurrence of severe complications (Clavien–Dindo III–V).

The finding of the current study is in line with previous studies. A systematic review with meta‐analysis demonstrated that low SMM was associated with the occurrence of severe postoperative complications in patients with head and neck squamous cell carcinoma (OR 4.79, 95% CI: 2.52–9.11) [6]. Previously we evaluated postoperative complications among patients who had undergone reconstruction of segmental mandibular defects with free fibula flaps [3]. In another article low SMM in FRFF was found to be a predictor for postoperative complications (OR 2.0, 95% CI 1.1–3.8, *p* = 0.03) [12]. We observed that low SMM was a negative predictive factor for postoperative flap complications. A retrospective case–control study by Alwani et al. [8] evaluated complications among 168 HNC patients who received autologous free vascularized tissue reconstruction. Patients with low SMM had higher rates of complications, including pneumonia, venous thromboembolism, longer mechanical ventilation times, delirium, wound disruptions/fistula, and intensive care unit stays. Overall, these patients had higher rates of any postoperative complications and also flap‐specific complications [8]. In another study of 239 HNC patients who underwent free flap reconstruction, low SMM was a predictor of perioperative blood transfusion requirements [13]. In a cohort of 206 HNC patients 30.1% were discharged after free flap reconstruction to post–acute care facilities, including skilled nursing facilities, in‐patient rehabilitation facilities, and long‐term care hospitals, for extended support and recuperation beyond the immediate postoperative setting. Low SMM was found to be independently associated with discharge to the above mentioned post–acute care facilities [14].

In this cohort flap, dehiscence was particularly present in the low SMM group 44.7% versus 31.9% in the normal SMM group. In line with this finding is a study of patients undergoing total laryngectomy, where sarcopenia was found to be the sole predictive factor of any wound complication (OR, 7.54; 95% CI, 1.56–36.4) [15].

The pathophysiological mechanisms underlying the association between preoperative sarcopenia and the risk of postoperative complications have not been elucidated. SMM depletion is associated the production of anti‐inflammatory cytokines and adiponectin decreases and the production of pro‐inflammatory molecules, such as leptin, chemerin, resistin, tumor necrosis factor‐α, interleukin‐1 and ‐6 increases [16, 17]. Based on this mechanism, patients with sarcopenia are considered to be in a pro‐inflammatory state. The pro‐inflammatory state leads to a weakening of the immune system and poor wound healing after surgery, thereby exerting an impact on the risk of postoperative complications [18].

Postoperative complications can have devastating consequences for both functional and cosmetic outcomes and can have a serious psychological impact on patients. Assessment of SMM is an objective measure that can provide valuable information in the clinical setting. It can be used to predict postoperative outcomes and consequently aid in surgical decision making. For instance, as in whether or not to opt for a microvascular reconstruction or a reconstruction with a local flap, which entails less risk of wound dehiscence and shorter operative time. Selecting patients with low SMM for a local flap reconstruction can potentially reduce postoperative complications.

Several methods to increase skeletal muscle mass and to decrease systemic inflammation have been reported in the literature [19]. In a double‐blind, randomized placebo‐controlled trial by Rooks et al. bimagrumab treatment, a monoclonal antibody that blocks activin type II receptor (ActRII) to inhibit myostatin signaling and stimulate protein anabolism was added to optimized standard of care in community‐dwelling older adults with sarcopenia [20]. The results showed no difference in the improvement of physical function between bimagrumab versus placebo, although participants who received bimagrumab had an increased lean body mass and reduced fat mass versus participants who received placebo.

Physical activity is also effective at mitigating sarcopenia. Several studies have demonstrated that prehabilitation in patients undergoing major abdominal surgery, particularly with exercise programs, results in lower rates of postoperative morbidity [21]. However, these studies did not stratify for sarcopenia.

Optimizing nutritional status may be valuable in patients prior to surgery because this can potentially decrease systemic inflammation and promote better wound healing. An example is the area of immunonutrition supplements that contain arginine, omega‐3 fatty acids, and dietary nucleotides that modulate prostaglandin E2 production, decrease IL‐6 production, and promote T‐cell differentiation. Mueller et al. demonstrated that the use of immunonutrition for 5 days prior to salvage surgery among patients with HNC compared to a control group, caused a significant reduction in overall complications (35% vs. 58%, *p* = 0.027) and in overall length of hospital stay (median 6 vs. 17 days, *p* = < 0.001) [22]. A study by Aeberhard et al. found that the use of immunonutrition for 5 days prior to surgery was associated with a significant decrease in the occurrence of wound abscesses and orocutaneous or pharyngocutaneous fistulas compared to the control group (7.4% vs. 15.3%, OR = 0.30, *p* = 0.006) [23].

The combination of nutritional intervention and exercise has also been reported. A recent meta‐analysis of 42 RCTs compared multiple exercise intervention arms in 3728 older people with sarcopenia [24]. This analysis found that adding nutritional interventions to exercise had a larger effect on handgrip strength than exercise alone while showing a similar effect on other physical function measures to exercise alone.

Our study was limited due to its single‐institution and retrospective nature and therefore limited by the available medical documentation. Heterogeneity in terms of types of flaps, that is, only FRFF and FUFF, was limited (particularly when compared to other studies), but may have introduced bias with respect to flap complications. Although we found an association between low SMM and severe complications and also flap complications, we could not determine the odds of specific complications such as pneumonia, wound infections, delirium and flap failure due to the limited number of events.

SMM was assessed on cross‐sectional imaging at the level of C3 because of the availability of routinely performed head and neck CT and MRI and its high correlation with the most often used SMM assessment at the third lumbar vertebra (L3) [25, 26, 27]. Changes in SMM occur over time since cancer‐related skeletal muscle depletion is a continuous process. This study is limited to preoperative SMM at a single point in time, although the degree and effect of eventual perioperative change of SMM is unknown.

In conclusion, low preoperative SMM has a negative impact on the occurrence of FFAF‐related complications in patients undergoing an FAFF reconstruction for oral cavity cancer. It is also a negative impact factor for severe (CD ≥ III) postoperative complications. Identification of high‐risk patients by SMM assessment allows for alternative surgical treatment planning, for example, less extensive surgery and less complex reconstructions, pre‐ and perioperative management and counseling.

## Data Availability

The data that support the findings of this study are available from the corresponding author upon reasonable request.
